# Post-activation performance enhancement (PAPE) and taurine combination improves anaerobic performance in highly trained wrestlers: a double-blind, randomized, crossover study

**DOI:** 10.1080/15502783.2026.2673071

**Published:** 2026-05-11

**Authors:** Süleyman Bi̇lgi̇n, Yusuf Buzdağli, Murat Ozan, Umut Yilmaz, Çağrı Çi̇ydem, Cemre Didem Eyi̇pinar, Muhammet Raşit İnaç, Ufuk Han Bağaçli, Halil Uçar, Erdinç Şiktar, Mücahit Dursun

**Affiliations:** a Department of Coaching Education, Faculty of Sport Sciences, Adıyaman University, Adıyaman, Turkey; b Department of Coaching Education, Faculty of Sport Sciences, Erzurum Technical University, Erzurum, Turkey; c Department of Physical Education and Sports, Faculty of Sport Sciences, Atatürk University, Erzurum, Turkey; d Department of Coaching Education, Faculty of Sport Sciences, Hakkari University, Hakkari, Turkey; e Department of Physical Education and Sport, Faculty of Sport Sciences, Gaziantep University, Gaziantep, Turkey; f Winter Sports and Sports Sciences Institute, Atatürk University, Erzurum, Turkey; g Ministry of National Education, Necmettin Erbakan Science High School, Erzurum, Turkey; h Department of Physical Education and Sports, Faculty of Education, Inonu University, Malatya, Turkey; i Department of Coaching Education, Faculty of Sport Sciences, Atatürk University, Erzurum, Turkey; j Department of Sport Management, Faculty of Sport Sciences, Selçuk University, Konya, Turkey

**Keywords:** Muscle activation, wrestling, 2-aminoethanesulfonic acid, PAPE, taurine

## Abstract

**Background:**

PAPE is a physiological phenomenon that temporarily enhances muscular strength and responsiveness following high-intensity muscle activity. This study aimed to investigate the acute effects of a PAPE protocol combined with taurine supplementation on anaerobic performance, blood lactate levels, and neuromuscular fatigue in highly trained male wrestlers.

**Methods:**

Twenty elite male wrestlers participated in a double-blind, crossover design comprising three separate sessions: (i) control with no supplementation or PAPE protocol (CON), (ii) PAPE protocol with placebo (PAPE*PLA), and (iii) PAPE protocol with taurine supplementation (PAPE*TAU). In the PAPE*PLA and PAPE*TAU conditions, participants completed 3 sets of 8 repetitions of squat and hip thrust exercises at 85% of their one-repetition maximum. Either a taurine supplement or a sucrose placebo was administered 60 minutes before the protocol. Five minutes after the PAPE protocol, the Wingate anaerobic power (WanT) test was conducted, while CMJ tests were performed before (CMJ-pre), immediately after (CMJ-post), and five minutes following (CMJ-post-5) the WanT.

**Results:**

The PAPE*TAU condition resulted in significantly higher peak power output (16.76% increase; *p* = 0.048) and power relative to body mass (22.24% increase; *p* = 0.028) compared to PAPE*PLA and CON. Additionally, post-test blood lactate levels were significantly lower in the PAPE*TAU condition (*p* < 0.05), and CMJ performance was significantly better post-exercise (*p* < 0.05), indicating reduced neuromuscular fatigue.

**Conclusion:**

In conclusion, the combination of the PAPE protocol with taurine supplementation enhances anaerobic performance, supports neuromuscular function, and promotes metabolic recovery following high-intensity exercise. These findings suggest a synergistic effect that may benefit athletes engaged in explosive and anaerobic sports such as wrestling.

## 1. Introduction

Wrestling is a complex sport that requires high levels of physical endurance, strength, speed, and strategic thinking. Matches are typically characterised by repeated short-duration, high-intensity efforts, during which anaerobic energy systems are predominantly utilised [1,2]. To perform at their peak, athletes must improve their anaerobic capacity, explosive strength, and fatigue resistance. Accordingly, the integration of acute performance-enhancing strategies into training programs has become a topic of growing interest in sports science research [3,4]. One such strategy is Post-Activation Performance Enhancement (PAPE), a physiological mechanism that temporarily enhances muscular strength and responsiveness following high-intensity muscle activation [5]. This response is believed to result from mechanisms such as myosin light chain phosphorylation, increased motor unit recruitment, and improved neuromuscular efficiency [6,7]. Research indicates that PAPE can enhance short-term performance, particularly when applied through high-resistance exercises like squats, Olympic lifts, or ballistic movements [4,8]. For optimal effect, it is recommended that loads of 85–90% of one-repetition maximum (1RM) be used, followed by a rest period of 5–8 minutes [9]. Therefore, the development of wrestling-specific PAPE protocols tailored to these parameters may help maximise anaerobic performance and provide a competitive edge in high-intensity combat sports [2,10–13].

During high-intensity anaerobic exercise, elevated blood lactate and hydrogen ion (H⁺) concentrations reduce muscle cell pH, impairing contraction capacity and ATP production [14,15]. These metabolic disturbances contribute to a reduction in muscle force output, ultimately leading to fatigue [15]. The 30-second WanT is commonly used to assess anaerobic capacity; however, the accumulation of H⁺ during this test suppresses phosphocreatine and phosphofructokinase enzyme activity, thereby limiting glycolysis and accelerating fatigue onset [16,17]. As a result, evaluating changes in muscle function after the WanT is essential for understanding performance in high-intensity sports. The countermovement jump (CMJ) test has been widely used as a valid and reliable method to monitor neuromuscular fatigue (NMF), track training adaptations, and assess recovery status [18,19]. CMJ height has demonstrated excellent test-retest reliability (ICC > 0.90), supporting its use as a consistent marker of NMF [20]. A post-exercise decline in CMJ performance is commonly interpreted as a marker of acute neuromuscular fatigue [21,22].

Given these performance-limiting physiological mechanisms, there is growing interest in nutritional strategies that can mitigate fatigue and promote recovery. Among the various ergogenic aids used by athletes, taurine (TAU) has emerged as a promising supplement due to its multifaceted benefits on muscle function [23,24]. Taurine enhances muscle contractions by modulating intracellular calcium homoeostasis [25], delays fatigue through improved cellular hydration [26], and minimises exercise-induced muscle damage via its antioxidant properties [24]. Furthermore, it supports mitochondrial energy production, which is vital for sustained high-intensity efforts [27]. Previous studies involving the WanT and repeated sprint protocols have demonstrated that taurine supplementation can increase explosive power and delay the onset of fatigue [19,28]. These properties suggest that taurine may serve as a valuable tool in counteracting the metabolic and neuromuscular challenges associated with intense anaerobic exercise.

While PAPE enhances force production by acutely activating the neuromuscular system through neural and mechanical pathways [5], taurine (TAU) supplementation may support performance improvement via intracellular mechanisms that regulate muscle function, reduce fatigue, and increase contraction efficiency [29]. Specifically, TAU enhances calcium handling, stabilises cell membranes, and mitigates oxidative stress, which are all essential for maintaining muscular output under intense workloads. These cellular-level benefits may complement the neuromechanical advantages of PAPE. Furthermore, TAU is suggested to improve neuromuscular communication by enhancing motor unit synchronisation, potentially amplifying the motor unit activation initiated by PAPE [24,28]. This theoretical synergy could provide a dual-layered enhancement of neuromuscular and metabolic performance that is especially advantageous for explosive, anaerobic disciplines such as wrestling.

Although the individual effects of both PAPE and taurine on anaerobic performance have been well-documented, the literature remains limited regarding their concurrent application. Most existing research has evaluated their isolated benefits, leaving the potential additive or synergistic effects of combining these two interventions underexplored. This gap is particularly relevant for elite athletes, where marginal gains in performance and recovery can yield substantial competitive advantages. Therefore, this study aimed to investigate the acute effects of combining a PAPE protocol with taurine supplementation on anaerobic performance, neuromuscular fatigue, and metabolic responses in highly trained wrestlers using a double-blind, randomised, crossover design. We hypothesised that combining PAPE with taurine supplementation would result in a superior acute enhancement of anaerobic power, neuromuscular performance, and recovery dynamics compared to either PAPE alone or no intervention.

## 2. Materials and methods

### 2.1. Participants

**Figure 1.**
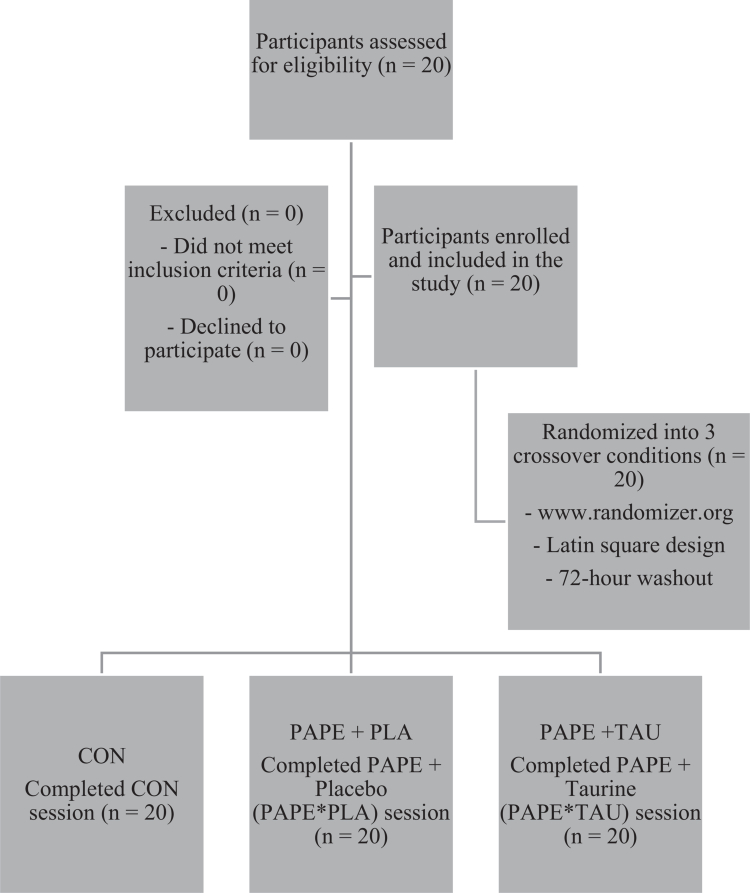
CONSORT diagram showing participant flow through the study.

A priori power analysis was conducted using G*Power 3.1 to determine the minimum required sample size for a repeated-measures design. The analysis indicated that 18 participants would be sufficient to detect a medium-to-large effect size (f = 0.30) with 80% power at an alpha level of 0.05. The sample size estimation was based on the primary outcome variable, peak power (PP), obtained from the Wingate Anaerobic Test (WanT), as it directly reflects anaerobic performance capacity and aligns with the main objective of the study. The anticipated effect size (f = 0.30) was selected based on previous crossover and intervention studies reporting moderate-to-large effects on anaerobic performance and related neuromuscular outcomes following taurine supplementation and PAPE protocols (Buzdağlı et al., 2023; Seitz & Haff, 2016; Wilson et al., 2013). To account for possible attrition and ensure robustness, 20 participants were recruited. This study included 20 highly trained male wrestlers with a mean age of 22.5 ± 4.3 years, body mass of 76.4 ± 7.5 kg, height of 177.7 ± 8.6 cm, and body mass index (BMI) of 24.0 ± 5.1 kg/m². Inclusion criteria required participants to have at least 10 years of training experience, achievements at the national or international level, and a minimum of three years of regular resistance training. Participants were selected among athletes who consistently completed their weekly training programs and had been engaged in high-intensity resistance training, particularly compound exercises such as hip thrusts and squats, for at least two years. Participant recruitment and flow through the study were strictly monitored and documented according to CONSORT guidelines, ensuring transparency and methodological rigour (Figure 1).

Descriptive data showed no notable differences between national- and international-level athletes in terms of age, anthropometric measurements, or baseline performance values. The comparability of these subgroups was considered sufficient to maintain internal consistency across the sample. Exclusion criteria included the use of any supplements, steroids, or doping substances that could affect hormone levels or athletic performance within the past three months; use of narcotic or psychotropic drugs; consumption of stimulants or banned agents during the study period; and the presence of cardiovascular, metabolic, neurological, pulmonary, orthopaedic, or neuromuscular disorders. Individuals without prior experience in high-intensity exercise, with regular sleep disorders, or who used addictive substances were also excluded. Moreover, those who had regularly used TAU or other neurotrophic supplements in the past three months were not eligible for participation.

Participants’ training, nutrition, and sleep routines were standardised for internal validity. Macronutrients (carbohydrates, protein, fat) and total energy intake data from dietary records were obtained using the TURKOMP food composition database (http://www.turkomp.gov.tr/database). Participants recorded a 24-hour dietary recall, which a sports dietitian evaluated. Daily macronutrient intake was summarised in Excel, and nutritional standardisation helped control metabolic variability throughout the study.

### 2.2. Study design

This study employed a double-blind, crossover design to minimise potential bias arising from the interaction between TAU intake and circadian rhythms. Participants attended three separate testing sessions, each separated by 72 hours (±0.5 hours), and visited the laboratory a total of four times. Using random assignment via www.randomizer.org, participants were allocated to one of three conditions: control (CON), taurine supplementation (TAU), or placebo (PLA). To maintain the integrity of the double-blind procedure, both participants and performance assessors were blinded to group allocation. The taurine and placebo solutions were prepared in identical volumes, taste, and appearance by an independent researcher who was not involved in any testing, data collection, or analysis. Blinding was sustained until all data were collected and statistical procedures were finalised.

**Figure 2.**
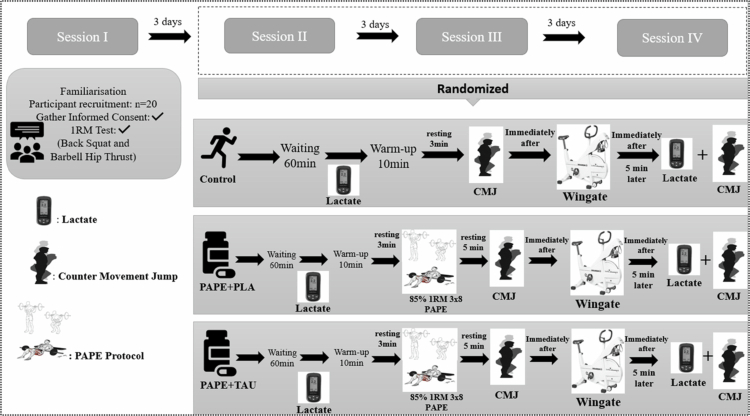
Visualisation of the experimental procedure.

During the first experimental session, participants were thoroughly informed about the study procedures, provided written informed consent, and underwent a one-repetition maximum (1RM) estimation test to determine maximal strength. The second, third, and fourth sessions were conducted in a randomised order and followed the protocol outlined in the Study Flow Diagram (Figure 2).

### 2.3. Experimental conditions and test protocols

*Control Session:* Participants rested for 60 minutes, followed by a standardised 10-minute warm-up protocol. After the warm-up, a 3-minute rest was given before administering the WanT. Additionally, to assess neuromuscular fatigue (NMF), the CMJ test was performed immediately before, immediately after, and five minutes after the WanT.

*PAPE Protocol:* In the PAPE protocol, participants performed squats and hip thrusts at 85% of their one-repetition maximum (1RM), completing three sets of 8 repetitions for each exercise. A 3-minute rest period was provided between sets. Five minutes after the completion of the PAPE protocol, the WanT was conducted. Similar to the control session, the CMJ test was administered immediately before, immediately after, and five minutes after the WanT to evaluate NMF.

PAPE*PLA and PAPE*TAU Sessions: Participants received a PLA or TAU supplement 60 minutes before testing in these sessions. The PAPE protocol followed a standardised 10-minute warm-up and 3-minute rest period. To assess NMF, the CMJ test was administered immediately before, immediately after, and five minutes after the WanT.

### 2.4. Supplementations and dietary control

Participants were instructed to arrive at the laboratory 90 minutes before initiating the exercise protocols. Based on random group assignment, they received either a TAU supplement (6 mg/kg) or a PLA (6 mg/kg sucrose). Considering that TAU plasma concentrations begin to rise approximately 10 minutes after ingestion and peak around 60 minutes, the supplement was administered 60 minutes before testing.

Participants were instructed to arrive at the laboratory 90 minutes prior to the initiation of exercise protocols to allow sufficient time for supplementation absorption and pre-test preparations. According to the randomised group allocation, participants received either a taurine (TAU) supplement at a dosage of 6 mg/kg body weight or an isocaloric placebo (PLA) consisting of 6 mg/kg sucrose. This dosage was selected based on prior studies demonstrating its efficacy in elevating plasma taurine concentrations to ergogenically relevant levels without adverse effects [24,27]. Pharmacokinetic data indicate that plasma taurine levels begin to rise approximately 10 minutes after oral ingestion and typically peak around 60 minutes [24]. Therefore, the supplement was administered exactly 60 minutes before testing to coincide with peak plasma concentration, aiming to maximise taurine’s potential physiological effects during the anaerobic exercise. This timing was chosen to optimise taurine’s roles in calcium handling, antioxidant activity, and neuromuscular function during performance.

To eliminate inter-individual dietary variability and standardise metabolic conditions across all test sessions, a detailed nutritional protocol was implemented. Each participant received an individualised diet plan with a macronutrient composition of 60% carbohydrates, 30% fats, and 10% protein, initiated 72 hours before the first experimental session.

Participants were explicitly instructed to replicate their pre-test meals, both in composition and timing, before each subsequent session to ensure intra-individual consistency. To verify dietary adherence, participants completed 24-hour food recall forms before each test session. These records were analysed by a certified sports dietitian using the TURKOMP food composition database (http://www.turkomp.gov.tr/database), and data were recorded in a standardised Excel spreadsheet to confirm macronutrient and caloric equivalence across sessions. Any deviations were flagged and addressed.

Given the potential of caffeine and other stimulants to interfere with neuromuscular and metabolic outcomes, strict abstention was required for at least 72 hours before each test session. A comprehensive list of caffeine-containing products, including coffee, tea, chocolate, cola beverages, energy drinks, and pre-workout supplements, was provided. Participants signed compliance declarations confirming adherence to this restriction. To control for residual effects of the interventions, a 72-hour washout period (±0.5 hours) was enforced between conditions. This was based on the reported elimination half-life of taurine (approximately 6.5 hours), ensuring that circulating levels returned to baseline before subsequent supplementation. This temporal gap was also intended to mitigate lingering neuromuscular fatigue or hormonal changes that could confound the outcomes. All supplementation and dietary standardisation procedures were implemented under supervision, and no major protocol deviations were identified throughout the study period.

### 2.5. Rating of perceived exertion (RPE)

RPE, commonly referred to in the literature as the Borg Scale (6–20), is a valuable tool for subjectively monitoring an individual's exercise tolerance [30]. The Borg Scale allows participants to express their perceived exertion levels during exercise on a continuum ranging from “nothing at all” (6), “very, very light” (7–8), “very light” (9–10), “light” (11–12), “somewhat hard” (13–14), “hard” (15–16), “very hard” (17–18), “very, very hard” (19), to “maximal exertion” (20). This scale helps track an individual's progression toward maximal effort during exercise testing [31]. In this study, the Borg Scale was administered immediately after the WanT to assess perceived exertion levels.

### 2.6. Blood lactate

Blood lactate levels were measured at three time points [32]: at rest (baseline), immediately after completing the WanT (L-post) (within approximately 1 minute post), and five minutes into the recovery phase (L-post-5). Blood samples (5 μL) were collected from the index fingertip of the left hand using the Lactate Scout 4 Sports analyser (Leipzig, Germany), following the manufacturer's instructions for proper handling and analysis.

### 2.7. Gastrointestinal symptom rating scale (GSRS)

**Figure 3.**
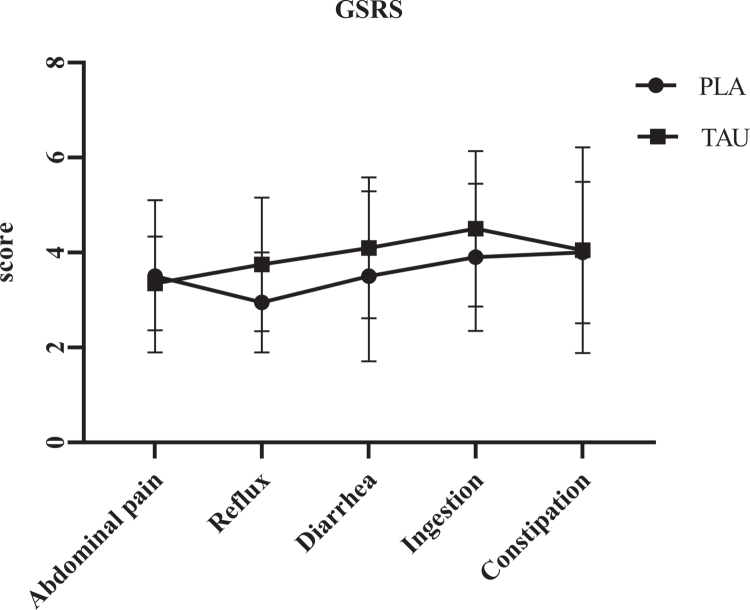
GSRS subscales in PLA and TAU supplement intake (*p* > 0.05).

GSRS was developed by Revicki, Wood [33] to assess common symptoms associated with gastrointestinal disorders. The scale consists of 15 items rated on a 5-point Likert scale, ranging from “no discomfort” to “very severe discomfort.” Items are grouped into five subscales: abdominal pain, reflux, diarrhoea, indigestion, and constipation. The total score ranges from 15 to 105, with higher scores indicating more severe symptoms. In this study, the GSRS was administered 24 hours after the intervention to evaluate delayed gastrointestinal responses (Figure 3).

### 2.8. Strength measurement test (maximum repetition test)

The test measures an individual's maximum weight for a single repetition. Several methods exist for determining 1RM; however, the testing protocol must be clearly defined before conducting the test. Rest periods and repetition counts should be carefully documented to ensure consistency across subsequent tests. In cases where directly measuring 1RM is unsafe, impractical, or poses a high risk of injury, indirect methods can estimate 1RM. This study conducted a randomised 1RM assessment for hip thrust and squat exercises during the first session using www.randomizer.org. Participants underwent a standardised warm-up protocol, including joint mobility exercises, dynamic stretching, core activation drills, and specific preparatory exercises (e.g. glute bridge or squat), lasting 10 minutes. To ensure accuracy and reliability, each participant's maximum number of repetitions was restricted to 10 or fewer, following the guidelines proposed by Mayhew, Johnson [34]. The Brzycki [35] equation was used to estimate 1RM:

1RM=(Lifted Weight)/[1,0278−(Repetitions × 0,0278)]

### 2.9. Neuromuscular fatigue

CMJ performance was assessed before and after the WanT using a standardised protocol previously described in the literature [36]. Measurements were taken at three time points: baseline (CMJ-pre), immediately after WanT (CMJ-post), and five minutes after WanT (CMJ-post-5). Participants performed two CMJ trials at each time point, separated by a 30-second rest period, using a Microgate Optojump force platform (Mahopac, USA). The mean values of peak power (PP), total jump time (TT), and average power (AP) from the two trials were recorded. Peak power (PP) was automatically calculated by the system using the following equation [37]:

PP(Watts)=(60.7×Jump Height(cm))+(45.3×Body Mass(kg))–2055

In this study, CMJ performance was assessed at two distinct time points: immediately before and immediately after the WanT. This approach was employed to accurately capture acute neuromuscular fatigue (NMF) induced by high-intensity anaerobic exercise. The pre-test measurement served as a baseline reference for the participants’ neuromuscular status, while the post-test measurement allowed for the quantification of fatigue-induced performance decrements. Assessing CMJ at two different time points increases the sensitivity of within-subject comparisons and helps detect subtle neuromuscular changes that might be overlooked in single-time-point assessments. This protocol aligns with previous research emphasising the importance of pre- and post-exercise CMJ assessments for monitoring fatigue and recovery dynamics [18,19].

### 2.10. Wingate Anaerobic Test

The WanT was conducted in accordance with established protocols from prior research. Under standardised conditions, the test was performed on a Monark Classic Ergomedic 894E cycle ergometer (Vansbro, Sweden). Before the test, seat height and foot positioning were carefully adjusted to ensure a near-full knee extension at the lowest pedal position. These settings were recorded and replicated for each test session to maintain consistency across all conditions. The resistance load was set at 7.5% of each participant’s body mass (kg), following standard WanT procedures. Before the test began, participants performed a short, maximal pedalling effort (without resistance) to accelerate the flywheel. Once maximum cadence was reached, the pre-determined resistance was applied, and participants were instructed to exert an all-out effort for 30 seconds. Standardised verbal encouragement was provided throughout the test to maximise participant motivation and performance.

Power output (W) was recorded every second during the 30-second WanT. From this data, four performance variables were analysed: Peak Power Output, the highest value recorded; Time to Peak Power, the time taken to reach this peak; Minimum Power Output, the lowest value in the final 10 seconds; and Average Power Output, the average power over the entire test duration [38].

### 2.11. Familiarisation

All participants included in the study were selected according to predefined inclusion criteria within the scope of the research project. Since the participants were highly trained athletes, they were already familiar with the WanT protocol. However, to ensure consistency and minimise potential variability, all participants were thoroughly informed about the testing procedures, exercise protocols, and expected performance requirements before the study commenced. All participants underwent a familiarisation session three days before the experimental protocol to enhance familiarity and eliminate potential learning effects. During this session, the WanT protocol was performed under identical conditions to the main testing phase, allowing participants to experience the workload, resistance settings, and pacing strategy in a controlled environment. A predefined, visualised experimental flowchart was developed to systematically outline the study design and procedural steps. This ensured standardisation of all experimental conditions, including participant preparation, supplementation timing, warm-up routines, and data collection procedures. All experimental procedures, including the detailed implementation and testing phases, were designed to maximise reproducibility and data reliability and are summarised accordingly in the study framework.

### 2.12. Statistical analysis

The data are presented as mean ± standard deviation (SD) and 95% confidence intervals (CI). Statistical significance was set at *p* < 0.05. Due to the sample size, the Shapiro–Wilk test was used to assess the normality of the data [39]. A two-way repeated-measures analysis of variance (ANOVA-RM) was applied for all variables, with condition and time as within-subject factors. The assumption of sphericity was evaluated using Mauchly’s test. When the sphericity assumption was violated (*p* < 0.05), Epsilon (*ε*) values were used to adjust the degrees of freedom, with the Greenhouse–Geisser correction applied when *ε* < 0.75 and the Huynh–Feldt correction applied when ε ≥ 0.75. When significant main effects were observed, Bonferroni-adjusted post-hoc pairwise comparisons were conducted to identify specific differences between conditions. When significant main or interaction effects were observed, Bonferroni-adjusted post-hoc pairwise comparisons were conducted to identify specific differences between conditions and/or time points. Effect sizes were calculated using partial eta squared (ηp²) and interpreted as small (0.01), moderate (0.06), and large (0.14) according to conventional thresholds [40]. Percentage changes were calculated to compare relative differences between the PAPE*PLA and PAPE*TAU conditions for selected performance variables. These comparisons were included to provide descriptive context for the magnitude of differences and were interpreted alongside the inferential statistical results. All statistical analyses were conducted using IBM SPSS Statistics for Windows, Version 25.0 (IBM Corp., Armonk, NY, USA). Visual representations were created using BioRender (https://biorender.com/) and GraphPad Prism (GraphPad Software, San Diego, CA, USA).

## 3. Results

The demographic and nutritional characteristics of the male athletes (*n* = 20) who participated in the study are presented in Table 1. The mean age of the participants was 23.9 ± 2.8 years [95% CI: 21.2, 26.5], and their average training experience was 14.7 ± 5.2 years [95% CI: 12.3, 17.1]. The athletes' mean body mass was recorded as 77.5 ± 7.2 kg [95% CI: 74.1, 80.8], height as 178.3 ± 6.4 cm [95% CI: 175.2, 181.3], and body mass index (BMI) as 24.5 ± 4.4 kg/m² [95% CI: 22.4, 26.5].

**Table 1.** Participant characteristics.

Variables	Male athletes (*n* = 20)	
	mean ± SD	[95% CI]
Age (years)	23.9 ± 2.8	[21.2, 26.5]
Training experience (years)	14.7 ± 5.2	[12.3, 17.1]
Body mass (kg)	77.5 ± 7.2	[74.1, 80.8]
Height (cm)	178,3 ± 6.4	[175.2, 181.3]
BMI (kg/m^2^)	24.5 ± 4.4	[22.4, 26.5]
Total energy intake (kcal/day)	3100	[2913, 3286]
Carbohydrate intake (g.kg^−1^/day)	4.2 ± 0.1	[4.2, 4.3]
Protein intake (g.kg^−1^/day)	2.4 ± 0.1	[2.3, 2.4]
Fat intake (g.kg^−1^/day)	1.0 ± 0.5	[0.7, 1.2]

Abbreviations: The mean and standard deviation [95% CI] are used to represent the data. BMI: Body Mass Index, kcal: kilocalories; g/kg^−1^: grams per kilogram of body mass.

The participants' average daily total energy intake was found to be 3100 kcal [95% CI: 2913, 3286]. Macronutrient intakes normalised to body mass were reported as 4.2 ± 0.1 g/kg for carbohydrates [95% CI: 4.2, 4.3], 2.4 ± 0.1 g/kg for protein [95% CI: 2.3, 2.4], and 1.0 ± 0.5 g/kg for fat [95% CI: 0.7, 1.2].

### 3.1. WanT performance

A significant main effect of condition was found for peak power (PP) (F = 2.745, *p* = 0.048, ηp² = 0.489). Bonferroni-adjusted post-hoc comparisons indicated that PP was significantly higher in the PAPE*TAU condition (1191.95 ± 288.54 W; 95% CI: 1056, 1326) compared to both CON (994.85 ± 231.49 W; 95% CI: 886, 1103) and PAPE*PLA (1020.25 ± 157.18 W; 95% CI: 946, 1093) (*p* < 0.05), while values were similar between CON and PAPE*PLA (*p* > 0.05). A significant main effect was also observed for peak power relative to body mass (PP W/kg) (F = 1.515, *p* = 0.028, ηp² = 0.521), with PAPETAU (14.73 ± 3.50 W/kg; 95% CI: 13.09, 16.37) exceeding both CON (11.65 ± 2.93 W/kg; 95% CI: 10.28, 13.02) and PAPEPLA (12.05 ± 2.64 W/kg; 95% CI: 10.81, 13.29) (*p* < 0.05). No significant differences were observed between conditions for average power (AP) (F = 1.932, *p* = 0.214, ηp² = 0.253) or minimum power (MP) (F = 1.428, *p* = 0.329, ηp² = 0.349), and post-hoc analyses confirmed that values were similar across all conditions (*p* > 0.05).

A significant effect of condition was found for time to peak power (T_PP) (F = 4.122, *p* < 0.001, ηp² = 0.550), with PAPETAU (1.75 ± 0.96 s; 95% CI: 0.55, 1.45) reaching peak power faster than both PAPEPLA (2.93 ± 1.01 s; 95% CI: 1.53, 2.47) and CON (3.72 ± 1.10 s; 95% CI: 2.49, 3.51) (*p* < 0.05). Additionally, PAPE*PLA demonstrated a shorter T_PP compared to CON (*p* < 0.05). RPE values were similar across all conditions (F = 1.453, *p* = 0.455, ηp² = 0.160), indicating that neither supplementation nor activation strategy influenced perceived exertion. Descriptive analyses showed that PAPETAU resulted in a 16.76% increase in PP, a 22.24% increase in PP W/kg, and a 40.27% reduction in T_PP relative to PAPE*PLA; however, these values are presented as complementary context to the statistically verified differences (Table 2).

**Table 2.** Physical, physiological, and perceptual responses under the experimental conditions.

Variables	CON Mean ± SD [95% CI]	PAPE*PLA Mean ± SD [95% CI]	PAPE*TAU Mean ± SD [95% CI]	Δ%	F	P	η_p_^2^
PP (W)	994.85 ± 231.49 [886, 1103]	1020.25 ± 157.18 [946, 1093]	1191.95 ± 288.54* [1056, 1326]	16.76	2.745	**0.048**	0.489
PP (W/kg)	11.65 ± 2.93 [10.28, 13.02]	12.05 ± 2.64 [10.81, 13.29]	14.73 ± 3.50* [13.09, 16.37]	22.24	1.515	**0.028**	0.521
AP (W)	638.85 ± 49.03 [615, 661]	642.40 ± 92.70 [599, 685]	659.11 ± 97.12 [613, 704]	2.64	1.932	0.214	0.253
MP (W)	344.05 ± 63.63 [314, 373]	351.71 ± 77.69 [315, 388]	393.50 ± 79.44 [356, 430]	11.96	1.428	0.329	0.349
T_PP (s)	3.72 ± 1.10 [2.49, 3.51]	2.93 ± 1.01* [1.53, 2.47]	1.75 ± 0.96*# [0.55, 1.45]	-40.27	4.122	**<0.001**	0.550
RPE	15.55 ± 2.21 [14.51, 16.58]	14.80 ± 2.23 [13.75, 15.84]	14.33 ± 2.28 [13.26, 15.40]	-3.17	1.453	0.455	0.160

Abbreviations: CON: control, PLA: placebo, TAU: taurine, PP: peak power, AP: Average power; MP: minimum power, T_PP: time to reach, RPE: the rating of perceived exertion, mean ± SD: mean ± standard deviation, 95% CI: 95% confidence interval, η_p_^2^_:_ partial eta square coefficient, *: Significantly different according to CON values (*P* < 0.05), ^#^: Significantly different according to PAPE*PLA values (*P* < 0.05). The percentage change was calculated between PAPE*PLA and PAPE*TAU conditions. Percentage change (Δ%) values represent the relative difference between the PAPE*TAU and PAPE*PLA conditions.

### 3.2. Blood lactate

**Figure 4.**
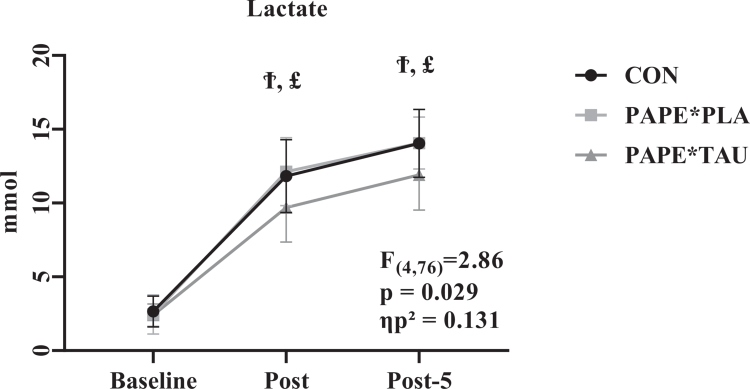
This figure shows the mean ± [95% CI] for blood lactate concentration registered at Lactate baseline, Lactate post, and Lactate post-5. **Ϯ**: significant differences between CON and PAPE*TAU, **£** : significant differences between PAPE*PLA and PAPE*TAU.

A significant condition × time interaction was observed for blood lactate responses (F_(4,76)_ = 2.86, *p* = 0.029, ηp² = 0.131), indicating that the temporal pattern of lactate responses differed across conditions. Bonferroni-adjusted post-hoc analyses revealed that Lactate post values were significantly lower in the PAPE*TAU condition (9.69 ± 2.32 mmol; 95% CI: 8.60, 10.78) compared to both CON (11.83 ± 2.46 mmol; 95% CI: 10.67, 12.98) and PAPE*PLA (12.12 ± 2.29 mmol; 95% CI: 11.04, 13.20) (*p* < 0.05), while values were similar between CON and PAPE*PLA (*p* > 0.05). Similarly, Lactate post-5 values were significantly lower in the PAPE*TAU condition (11.91 ± 2.37 mmol; 95% CI: 10.79, 13.02) compared to both CON (14.04 ± 2.29 mmol; 95% CI: 12.97, 15.12) and PAPE*PLA (14.06 ± 1.75 mmol; 95% CI: 13.23, 14.88) (*p* < 0.05), while no differences were observed between CON and PAPE*PLA (*p* > 0.05). These findings indicate that the PAPE*TAU condition was associated with a more favourable lactate response profile following high-intensity exercise, particularly during the recovery phase (Figure 4).

### 3.3. Neuromuscular fatigue

**Figure 5.**
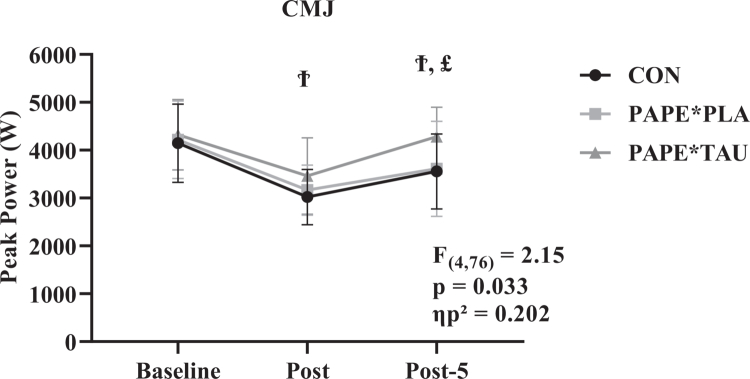
This figure shows the mean ± [95% CI] for CMJ registered at CMJ baseline, CMJ post, and CMJ post-5. **Ϯ**: significant differences between CON and PAPE*TAU, **£** : significant differences between PAPE*PLA and PAPE*TAU.

A significant condition × time interaction was observed for CMJ performance (F(4,76) = 2.15, *p* = 0.033, ηp² = 0.202), indicating that the temporal pattern of CMJ responses differed across conditions. Bonferroni-adjusted post-hoc analyses showed that CMJ post values were significantly higher in the PAPE*TAU condition (3461 ± 797 score; 95% CI: 3087, 3834) compared with CON (3020 ± 578 score; 95% CI: 2749, 3291) (*p* < 0.05), while values were similar between PAPE*PLA and CON (*p* > 0.05). Additionally, CMJ post-5 values were significantly higher in the PAPE*TAU condition (4281 ± 616 score; 95% CI: 3992, 4569) compared to both CON (3555 ± 783 score; 95% CI: 3188, 3922) and PAPE*PLA (3610 ± 990 score; 95% CI: 3146, 4074) (*p* < 0.05), while values were similar between CON and PAPE*PLA (*p* > 0.05). These findings suggest that the PAPE*TAU condition was associated with a more favourable neuromuscular performance profile during the post-exercise and early recovery phases (Figure 5).

## 4. Discussion

The primary aim of this study was to investigate the acute effects of a PAPE protocol combined with taurine supplementation on anaerobic performance, blood lactate responses, and neuromuscular outcomes in highly trained wrestlers. Importantly, the interpretation of CMJ and blood lactate responses was based on the condition × time interaction, which reflected differences in temporal response patterns across conditions. The main findings indicate that the PAPE*TAU condition was associated with a more favourable temporal response pattern in both metabolic and neuromuscular variables compared to the other experimental conditions. Importantly, the presence of significant condition × time interactions for both blood lactate and CMJ performance indicates that the magnitude and trajectory of responses differed across conditions. This suggests that the observed effects should be interpreted in terms of time-dependent changes rather than isolated condition differences. For anaerobic performance, the PAPE*TAU condition was associated with higher peak power output and a shorter time to peak power than the other conditions. These findings suggest that the combined application of taurine supplementation and PAPE may facilitate a more rapid expression of explosive force. However, these outcomes should be interpreted as performance-based observations rather than direct evidence of underlying neuromuscular mechanisms.

Expanding on these findings, the results from the WanT suggest that the combination of PAPE and taurine supplementation may be associated with a more favourable response in short-duration, high-intensity power output. The observed increases in peak power (PP) and body mass-normalised peak power (PP W/kg) in the PAPE*TAU condition indicate a greater capacity for rapid energy production compared with the other conditions. Moreover, the reduction in time to peak power (T_PP) suggests a faster expression of explosive force; however, this should be interpreted as a performance outcome rather than direct evidence of enhanced motor unit activation [3,4,41]. These findings are consistent with previous literature demonstrating the transient performance-enhancing effects of PAPE protocols [2,5,13,42]. In the present study, the use of approximately 85% of one-repetition maximum (1RM) loads combined with recovery intervals optimised for potentiation may have contributed to the observed performance responses. Nevertheless, these effects should be interpreted within the context of the study design and the measured outcomes.

Analysis of blood lactate responses showed that values were similar between the PAPE*PLA and control conditions at both post-exercise time points, suggesting that PAPE alone may have a limited influence on the temporal pattern of metabolic responses during high-intensity exercise. Previous studies have suggested that performance changes following PAPE are more closely associated with neuromuscular factors than with metabolic alterations [5,9]. In the present study, the absence of differences in lactate responses between the PAPE*PLA and control conditions is consistent with this perspective; however, these findings should be interpreted cautiously, as metabolic processes were not directly assessed.

Robbins [42] highlighted that the effectiveness of PAPE may depend on the appropriate selection of loading strategies and recovery intervals. In line with this, the lack of differences in lactate responses between the PAPE*PLA and control conditions in the present study suggests that PAPE alone may not substantially modify the temporal profile of metabolic stress under the current experimental conditions.

Recent studies suggest that neuromuscular adaptations, such as improved motor unit synchronisation and enhanced calcium handling, may contribute to the transient increases in force production observed following PAPE protocols [3,4]. However, the magnitude of these effects appears to vary depending on factors such as training status, activation intensity, and recovery duration [41]. Therefore, careful consideration of loading strategies and rest intervals may be necessary to optimise performance outcomes. Future research should further investigate these individual responses to better inform the application of PAPE in elite sports settings.

The performance improvements observed in this study suggest that taurine supplementation may be associated with enhanced anaerobic performance when combined with a PAPE protocol. Taurine is known to influence several physiological processes that could support high-intensity exercise performance, including contributing to membrane stabilisation and neuromuscular transmission [43,44]. In addition, evidence indicates that taurine may help regulate intracellular calcium dynamics by facilitating calcium release from the sarcoplasmic reticulum, potentially aiding rapid force production during intense muscular contractions [45]. These mechanisms were not directly assessed in the present study, but may partially explain the observed performance outcomes.

Taurine may also support performance during intense anaerobic exercise through its antioxidant properties, which may help reduce exercise-induced oxidative stress and support the maintenance of muscle function. These mechanisms were not directly measured in the present study; however, the lower post-exercise lactate values and higher CMJ performance observed in the PAPE*TAU condition may reflect a more favourable metabolic and neuromuscular response pattern during recovery. Previous research has similarly reported improvements in fatigue resistance and high-intensity performance following taurine supplementation [26,43,45,46], although such effects are not consistently observed across all populations or exercise modes [28,47]. Collectively, available findings indicate that taurine may be associated with an additional ergogenic contribution.

The improvements in CMJ performance observed in the PAPE*TAU condition suggest that taurine may be associated with the maintenance of neuromuscular performance. Previous research indicates that taurine has been reported to influence neuromuscular transmission [43,45]. Although the underlying physiological mechanisms were not directly assessed in the present study, these effects may partially explain the observed maintenance of jump performance.

In addition to its potential neuromuscular effects, taurine may also be associated with differences in metabolic responses. The lower post-exercise lactate values observed in the PAPE*TAU condition could indicate a more favourable metabolic response pattern for sustaining high-intensity efforts; however, the underlying mechanisms were not directly assessed in this study. These improvements, together with the enhanced CMJ performance, suggest that taurine supplementation may be associated with the maintenance of performance during the early recovery phase. Collectively, these findings imply that the combination of PAPE and taurine supplementation may represent a potentially beneficial acute ergogenic strategy for athletes requiring rapid power production and short recovery periods. Further research is warranted to clarify the mechanistic pathways and to determine whether similar benefits are observed across different populations and training contexts.

## 5. Conclusion

This study demonstrated that the application of a PAPE protocol combined with taurine supplementation was associated with significant acute effects on anaerobic performance, neuromuscular function, and metabolic responses in highly trained wrestlers. The findings indicate that this combination may be applicable for enhancing performance (particularly peak power and jump performance) and supporting short-term recovery responses.

However, the PAPE protocol alone was associated with limited and individually variable effects on certain performance parameters and did not appear to meaningfully influence the temporal pattern of metabolic responses. The addition of taurine supplementation may have contributed to these outcomes, potentially through mechanisms such as neuromuscular function, regulation of intracellular calcium, and antioxidant activity; however, these mechanisms were not directly assessed in the present study and should be interpreted with caution.

Given the variability in findings across the literature regarding taurine supplementation and the influence of factors such as training status, exercise modality, timing, and individual physiological responses, further research is warranted. In particular, randomised controlled trials across different athletic populations, exercise protocols, and dosing strategies are needed to improve the generalisability and practical applicability of these findings. Additionally, future studies should examine long-term effects and potential contributions to chronic training adaptations.

## 6. Strengths and limitations

This study presents several strengths that contribute to its scientific rigour and relevance. The use of a double-blind, randomised, crossover design minimises bias and allows each participant to serve as their own control, enhancing the internal validity of the findings. The inclusion of highly trained elite wrestlers increases the applicability of the results to high-performance athletes. Additionally, the comprehensive assessment of anaerobic performance through physiological (WanT, blood lactate) and neuromuscular (countermovement jump) measures provides a multidimensional understanding of the effects of PAPE in combination with taurine supplementation. The standardised protocols for supplementation, nutrition, and testing procedures further strengthen the consistency and reproducibility of the results.

Despite these strengths, some limitations should be acknowledged. The sample size, although consistent with similar intervention studies in elite athletes, remains relatively small and may limit the generalisability of the findings to broader athletic populations or other sports disciplines. The study’s acute design does not allow for assessment of long-term adaptations or chronic effects of combined PAPE and taurine supplementation. Furthermore, individual variability in response to supplementation and exercise protocols may have influenced the outcomes, and future studies could benefit from exploring these differences in greater detail. Finally, while we controlled for several confounding variables, other factors, such as psychological state or sleep quality, were not directly measured and could have impacted performance.

In conclusion, this study provides valuable insights into the acute ergogenic effects of combining PAPE protocols with taurine supplementation in elite wrestlers, while also highlighting areas for future research to further elucidate these interactions.

## 7. Practical applications

- Integrating taurine supplementation into a PAPE protocol may serve as an effective strategy to enhance peak power output and support neuromuscular performance, particularly in short-duration, high-intensity sports such as wrestling, sprinting, or weightlifting.
- When applied before exercise, this combined approach can be used to improve explosive performance during testing or as part of pre-competition warm-up routines.
- Due to its potential to attenuate lactate accumulation, taurine may contribute to reduced muscle acidosis and faster post-exercise recovery.
- This protocol may offer a competitive advantage during multi-bout competitions or tournaments that require repeated high-intensity efforts with limited recovery time.
- Coaches and performance specialists should tailor the timing (e.g. 4–6 minutes activation period) and dosing based on individual responses to maximise effectiveness.
